# Factors affecting hallucinations in patients with delirium

**DOI:** 10.1038/s41598-021-92578-1

**Published:** 2021-06-21

**Authors:** Masako Tachibana, Toshiya Inada, Masaru Ichida, Norio Ozaki

**Affiliations:** 1 Department of Psychiatry, Nagoya Ekisaikai Hospital, 4-66, Shonen-cho, Nakagawa-ku, Nagoya-shi, Aichi 454-0854 Japan; 2 Department of Psychiatry, Nagoya University Graduate School of Medicine, 65 Tsurumai-cho, Showa-ku, Nagoya-shi, Aichi 466-8550 Japan; 3 Department of Psychobiology, Nagoya University Graduate School of Medicine, 65 Tsurumai-cho, Showa-ku, Nagoya-shi, Aichi 466-8550 Japan

**Keywords:** Medical research, Risk factors

## Abstract

Delirium develops through a multifactorial process and include multiple subtypes with different pathological factors. To refine the treatment and care for delirium, a more detailed examination of these subtypes is needed. Therefore, this study aimed to explore the factors affecting delirium in cases in which hallucinations are conspicuous. In total, 602 delirium cases referred to the psychiatry department at a general hospital between May 2015 and August 2020 were enrolled. The Delirium Rating Scale-revised-98 was used to assess perceptual disturbances and hallucinations in patients with delirium. Multiple regression analysis was applied to determine whether individual factors were associated with the hallucinations. A total of 156 patients with delirium (25.9%) experienced hallucinations, with visual hallucinations being the most common subtype. Alcohol drinking (*p* < 0.0005), benzodiazepine withdrawal (*p* = 0.004), and the use of angiotensin II receptor blockers (*p* = 0.007) or dopamine receptor agonists (*p* = 0.014) were found to be significantly associated with hallucinations in patients with delirium. The four factors detected in this study could all be reversible contributing factors derived from the use of or withdrawal from exogenous substances.

## Introduction

Delirium is often multifactorial in etiology and can be influenced by several factors^1,2^. Delirium is defined as a disturbance in attention and awareness (reduced orientation to the environment) in the Diagnostic and Statistical Manual of Mental Disorders, Fifth Edition (DSM-5)^3^. Although the inter-rater reliability of a diagnosis of delirium is expected to increase because of the simple definition in the DSM-5, based on the diagnostic criteria, delirium includes multiple subtypes with different pathological factors. However, the ways in which certain subtypes of delirium may be affected by various factors have not been adequately investigated. To refine the treatment and care for delirium, a more detailed examination of these subtypes is needed.

Therefore, this study aimed to explore the precipitating factors of delirium according to cross-sectional symptoms, focusing especially on hallucinations among the various psychiatric symptoms observed in delirium. Hallucinations are perception-like experiences that occur without an external stimulus^3^. Hallucinations in delirium can be frightening and may cause emotional distress to the patients^4,5^, as well as their family members, caregivers, and nurses^6^. However, little remains known about the prevalence, risk factors, and phenomenology of hallucinations in delirium.

Therefore, in this study, to help provide a clinical subdivision of delirium that could lead to more appropriate treatment and care in clinical practice, various factors affecting the development of hallucinations in patients with delirium, including demographic, clinical, and pharmacological aspects, were examined.

## Methods

### Procedure

A survey was conducted on inpatients at Nagoya Ekisaikai Hospital, a general hospital with 602 beds (54 in the emergency center and no psychiatric beds, and no geriatric department). The targeted participants were 602 patients with delirium who had been referred to the psychiatry department at a general hospital from May 2015 to August 2020. Patients who had a history of schizophrenia were excluded. Delirium was diagnosed according to the DSM-5 criteria^3^ by two full-time trained psychiatrists. The following clinical variables were extracted from the medical records: sex, age, complications of visual and hearing impairment, eye disease (e.g., cataracts, diabetic retinopathy, glaucoma, other) and treatment, dementia (dementia with Lewy bodies and others), cerebrovascular disorder, Parkinson’s disease before admission, a history of psychiatric illness before admission, recent alcohol consumption before admission (habitual, heavy, dependent), a history of chronic smoking before admission, whether patients had stopped taking benzodiazepines (BZDs) after admission (BZD withdrawal), presence or absence of acute brain disorder and of infection, presence or absence of surgery (cardiac surgery and non-cardiac surgery), general anesthesia, oral ingestion ability, whether patients had been in an intensive care unit (ICU) during the development of delirium, pharmacological treatment after admission, and perceptual disturbances and hallucinations as assessed using the Japanese version of the Delirium Rating Scale-revised-98 (DRS-R-98)^7^ on the day when they were at their most severe level. Assessments were based on all available sources of information, including medical records, nurses, the attending physician, other medical staff, family members, and visitors. The severity items for perceptual disturbances and hallucinations on the DRS-R-98 were graded into 4 levels from normal (0) to severely impaired: hallucinations (3). The pharmacological treatment variable included corticosteroids, opioid analgesics, anticholinergics, histamine 1 or 2 receptor blockers, calcium channel blockers, β-blockers, angiotensin-converting enzyme (ACE) inhibitors, angiotensin II receptor blockers (ARBs), anti-arrhythmic drugs, digitalis, dopamine receptor agonists, nonsteroidal anti-inflammatory drugs, antibiotics, anticonvulsants, cholinesterase inhibitors, and hypnotics (e.g., BZDs, suvorexant, ramelteon). These include (1) medications that the patients had been receiving before hospitalization and continued receiving after hospitalization, and (2) those newly prescribed that the patients were receiving after hospitalization. The above variables used to examine their association with perceptual disturbances and hallucinations in the patients with delirium were chosen on the basis of previous reports and clinical relevance. Severity of alcohol consumption was defined as follows: 0, none; 1, habitual, i.e., patients who drink 1–3 units of alcohol per day; 2, heavy, i.e., patients who drink more than 4 units per day, but are not satisfied with the diagnosis of dependence according to the DSM-5 criteria; 3, dependence, i.e., patients who drink more than 4 units per day, and are diagnosed according to the DSM-5 criteria by psychiatrists before or after admission.

### Statistical analysis

As statistical analyses, multiple stepwise regression analysis were performed using the IBM SPSS statistical software ver. 27.0 (IBM Corp., Armonk, NY, USA). Significant difference was set at *p* < 0.05. The above all collected variables have been tested in multivariate analysis.

### Ethics statement

This study was initiated after the approval of the ethics committee of the Nagoya Ekisaikai Hospital and carried out in accordance with the Helsinki Declaration of 1964 and its later versions. Since the informed consent cannot be obtained from the patients who have developed delirium, the present study was conducted using an opt-out system for the use of anonymous patient data for scientific research.The right to opt out was informed for all participants.

### Consent to participate

The right to opt out was informed for all participants.

### Consent for publication

All authors agreed to publish this manuscript.

## Results

### Demographic and clinical characteristics of the participants

Of the 602 patients who developed delirium, 341 (57%) were male and 261 (43%) were female. The average age was 75.9 years (standard deviation, 12.2; age range, 24.3–100.9 years). The numbers of medical complications were as follows: visual impairment, 265 (44%); hearing impairment, 148 (25%); habitual alcohol drinking [dependent, 43 (7%); heavy, 41 (7%); habitual, 132 (22%)]; and a history of chronic smoking, 294 (49%). The participants’ demographic and clinical characteristics are shown in Table 1, and the prescribed medications are shown in Table 2.

**Table 1:** Demographic and clinical characteristics of the patients with delirium.

Demographic and clinical variables	Total	Perceptual disturbance and hallucinations (DRS-R-98): 0–3			
		Not present	Mild perceptual disturbance	Illusions present	Hallucinations present
Number of patients (%)	602				
Male ratio [percent]	57%	55%	61%	37%	56%
Mean age [years] (SD)	75.9 (12.2)	78.0 (11.7)	75.2 (12.2)	76.9 (10.8)	74.2 (12.9)
Rate of patients drinking alcohol					
Habitual	22%	22%	24%	15%	19%
Heavy	7%	5%	5%	11%	10%
Dependence	7%	1%	8%	7%	12%
Rate of patients with the following complications					
Chronic smoking	49%	44%	51%	33%	53%
Visual impairment	44%	44%	44%	26%	46%
Eye diseases					
Cataract	16%	16%	14%	7%	20%
Diabetic retinopathy	12%	12%	13%	11%	9%
Glaucoma	5%	5%	4%	11%	8%
Other eye diseases	10%	10%	8%	7%	13%
Rate of patients receiving treatment for eye diseases (e.g., eye drops)	13%	13%	12%	11%	15%
Hearing impairment	25%	27%	24%	26%	22%
Cerebrovascular disorder	19%	17%	19%	15%	20%
Parkinson's disease	2%	1%	2%	0%	4%
Dementia with Lewy bodies (DLB)	1%	0%	2%	4%	2%
Dementia excluding DLB	46%	46%	51%	37%	39%
Psychiatric diagnosis before admission	14%	12%	15%	15%	13%
Acute brain disorder before delirium developed during hospitalization	17%	14%	19%	16%	16%
Infection before delirium developed during hospitalization	19%	17%	22%	33%	14%
Rate of patients who have stopped taking BZDs after admission (BZD withdrawal)	21%	15%	22%	22%	28%
Rate of patients receiving cardiac surgery before delirium developed during hospitalization	8%	8%	11%	0%	5%
Rate of patients receiving non-cardiac surgery before delirium developed during hospitalization	21%	29%	15%	22%	23%
Rate of patients receiving general anesthesia before delirium developed during hospitalization	25%	33%	22%	7%	22%
Rate of patients who were in intensive care units when delirium developed	51%	43%	58%	48%	52%
Status of oral ingestion ability					
Patients in whom oral ingestion was possible	69%	69%	65%	85%	72%
Patients in whom tube feeding was available	10%	8%	12%	4%	10%
Patients in whom tube feeding was unavailable	21%	23%	23%	11%	18%
Laboratory findings					
Average level of Alb [g/dL] (SD)	3.0 (0.7)	3.0 (0.7)	3.0 (0.6)	3.0 (0.7)	3.1 (0.7)
Average level of Hb [g/dL] (SD)	11.2 (2.4)	11.0 (2.3)	11.1 (2.4)	11.1 (2.9)	11.5 (2.4)

SD, standard deviation; BZD, benzodiazepine; Ab, albumin; Hb, hemogrobin; DLB, dementia with Lewy body.

**Table 2:** Prescribed medications in patients with delirium.

Type of medication	Total (%)	Perceptual disturbance and hallucinations (DRS-R-98): 0–3			
		Not present (%)	Mild perceptual disturbance (%)	Illusions present (%)	Hallucinations present (%)
Corticosteroids	12	15	10	7	13
Opioid analgesics	22	21	25	26	19
Anticholinergics^a^	25	20	28	22	26
Histamine 1 (H_1_) receptor blockers	7	11	6	4	6
Histamine 2 (H_2_) receptor blockers	12	15	11	11	11
Calcium (Ca) channel blockers	36	37	36	26	38
β-blockers	21	20	23	22	17
Angiotensin-converting enzyme inhibitors	6	3	12	0	2
Angiotensin II receptor blockers	14	11	13	19	19
Antiarrhythmic drugs^b^	4	5	3	4	5
Digitalis	1	2	1	0	2
Dopamine receptor agonists	2	2	1	0	7
Nonsteroidal anti-inflammatory drugs	29	35	24	26	28
Antibiotics	48	47	51	56	43
Anticonvulsants	4	2	5	4	6
Cholinesterase inhibitors	6	5	8	7	4
Benzodiazepines	58	56	61	63	54
Suvorexant	18	15	19	7	21
Ramelteon	11	7	12	11	12

^a^Compounds that have anticholinergic activity, such as H1 blockers, H2 blockers, and dopamine receptor agonists, are not included in the "anticholinergics".

^b^Compounds that have antiarrhythmic activity, such as b-blockers and calcium channel blockers, are not included in the "antiarrhythmic drugs".

### Prevalence of hallucinations

Of the 602 patients with delirium, 156 (25.9%) experienced hallucinations during hospitalization, 27 (4.5%) experienced illusions, 241 (40.0%) experienced mild perceptual disturbances, and 178 (29.6%) did not experience any perceptual disturbances or hallucinations (Table 1).

### Pathological features of hallucinations

Of the 156 patients who experienced hallucinations, 144 (92.3%) experienced visual hallucinations. The other types of hallucinations experienced in delirium were auditory (n = 26, 16.6%), tactile (n = 3, 1.9%), and olfactory (n = 1, 0.6%) hallucinations. Of these, 17 patients (10.9%) experienced two or more hallucination subtypes simultaneously.

Most of the visual hallucinations experienced by patients with delirium were complex and concrete, such as people (family and friends, n = 35; strangers, n = 61; Buddha, n = 2; the devil, n = 2; ghosts, n = 4; cadavers, n = 2); insects, n = 22; animals, n = 20; inorganic objects, n = 15; and visions, n = 26 (fire, n = 15; falling ceilings, n = 5; flowing water, n = 3; quarrels and wars, n = 3). Of these, the visual hallucinations especially strangers or extraordinary visions such as fire caused substantial anxiety and confusion. One subject saw a stranger, saying “he was creepy.” There was one subject who experienced simple, elemental visual hallucination, saying “I saw a big color.”

### Factors affecting hallucinations in patients with delirium

Multivariate analysis determined that alcohol drinking (*p* = 0.000), BZD withdrawal (*p* = 0.004), and the use of ARBs (*p* = 0.007) or dopamine receptor agonists (*p* = 0.014) were significantly associated with hallucinations in patients with delirium as assessed by the DRS‐R98 (Table 3). No other variables were found to be associated with hallucinations in patients with delirium. The ARB and dopamine receptor agonist compounds are shown in Table 4. Of the group with hallucinations, 43 patients had stopped receiving BZDs after hospitalization, and 65% of them developed hallucinations within 2 days of BZDs discontinuation, and 91% within 6 days.

**Table 3:** Variables significantly associated with hallucinations in delirium.

Variables	Hallucinations in delirium		
	B	*p* value	95%CI
Alcohol drinking	0.219	0.000	0.119–0.319
Benzodiazepine (BZD) withdrawal	0.320	0.004	0.102–0.539
Use of angiotensin II receptor blockers	0.357	0.007	0.099–0.615
Use of dopamine receptor agonists	0.804	0.014	0.164–1.445

CI, confidence interval; BZD, benzodiazepine.

**Table 4:** Compounds of angiotensin II receptor blockers and dopamine receptor agonists.

Type of medication	Compound (number of patients prescribed)					
Angiotensin II receptor blockers	Azilsartan	(15)	Candesartan	(14)	Irbesartan	(6)
	Losartan	(6)	Olmesartan	(18)	Telmisartan	(19)
	Valsartan	(10)				
Dopamine receptor agonists	Amantadine	(2)	Bromocriptine	(1)	Levodopa	(9)
	Pramipexole	(2)	Ropinirole	(2)		

## Discussion

The results of this study revealed that 156 patients with delirium (25.9%) experienced hallucinations and 27 (4.5%) experienced illusions. Visual hallucinations were the most common subtype among all hallucinations. Alcohol drinking, BZD withdrawal, and the use of ARBs and dopamine receptor agonists were positively associated with hallucinations in patients with delirium, suggesting that these variables act as aggravating factors.

In a previous report evaluating the frequency of hallucinations with delirium assessed using the DRS-R-98, moderate (illusions present) or severe (hallucinations present) perceptual disturbances and hallucinations were reported in 26% of patients^8^. A similar prevalence was observed in the present study. Hallucinations in delirium have been reported to involve a high frequency of visual hallucinations^9^, but the content of such hallucinations has seldom been investigated. In a study on hallucinations after cardiac surgery, of 44 patients (delirium present: n = 10) who experienced hallucinations, complex and concrete visual hallucinations were reported in 31 (70.5%)^10^.

In the present study, alcohol drinking, BZD withdrawal, and use of ARBs and dopamine receptor agonists were significantly associated with hallucinations in patients with delirium; no associations were observed with intrinsic factors such as sensory impairment, eye diseases, psychiatric disorders, dementia and other neurological brain disorders, or environmental factors such as a stay in the ICU. In cases of delirium where hallucinations are conspicuous, the effects of exogenous substances, such as alcohol and drugs, should be prioritized, and appropriate treatment must be provided for each.

Numerous reports have been published on hallucinations caused by alcohol withdrawal^9^. On the other hand, few reports have been published on hallucinations caused by high- or low-dose BZD withdrawal^11–13^. BZDs are still frequently prescribed for insomnia and anxiety, not only in psychiatry^14,15^, but also in general medicine^16^, and many older patients are prescribed BZDs before hospital admission in Japan. In this study, BZD withdrawal (usual dose) was significantly associated with hallucinations in delirium. Although BZD withdrawal itself seldom results in the development of hallucinations^13^, the results of this study suggest that BZD withdrawal in patients with delirium is likely to lead to hallucinations.

There are few reports of hallucinations and delirium associated with angiotensin system inhibitors (ARBs and ACE inhibitors). In a previous report, visual hallucinations were reported after the use of ACE inhibitors^17^. On the other hand, ARBs have been reported to exhibit an inverse association with delirium^18^, and starting ACE inhibitors or ARBs postoperatively has been reported to be associated with reduced delirium^19^. In the present study, the use of ARBs, but not ACE inhibitors, was positively associated with hallucinations in patients with delirium. In a previous report of ACE inhibitors and hallucination, it is thought that older patients, particularly those with a history of either dementia or mild cognitive impairment, may be at higher risk for hallucination^17^. In the present study, in the group of patients receiving ACE inhibitor, the average age was 78.4 years and 39% had dementia, while in the group of patients receiving ARB, the average age was 80.7 years and 61% had dementia. These differences of patient background may have influenced the results. The present results suggest that ARBs may produce cases of delirium where hallucinations are conspicuous. Based on this finding, reducing the dose of ARBs or switching ARBs to other types of antihypertensive drugs could diminish the occurrence of hallucinations.

The use of dopamine receptor agonists in Parkinson’s disease is a difficult problem. Many of the hallucinations in Parkinson’s disease have been reported to be brief and nonthreatening; however, they tend to become worse at night or when vision is compromised; alterations in visual acuity are a particularly strong risk factor for visual hallucinations^20^. Tapering dopaminergic medication doses and using visual/hearing aids have been reported to be successful in the management of hallucinations with delirium in Parkinson’s disease. However, patients with Parkinson’s disease might not tolerate reductions in dopaminergic medication does and may therefore require compensatory increases in levodopa to prevent severe akinetic-rigid symptoms^20^.

This study did have several limitations. First, this was a retrospective study based on clinical practice at a single hospital. Further studies involving patients in other types of general hospitals could help confirm the present results. Second, since the delirium cases in this study were referral cases from other departments to the psychiatric department, most of delirium cases were classified as moderate or severe. Therefore, patients without delirium (healthy controls) or with only mild delirium who were not required to be referred to the psychiatric department were not included in the present study. To examine the exact association for all ranges of severity of hallucinations in patients with delirium, further research is needed involving patients without or with only mild delirium. Third, the patients in this study were receiving various kinds of medications, some of which were not included in the present analysis. More detailed research, such as that into drug interactions and dose-finding effects, is needed, including the various medications not investigated in the present study. Fourth, regarding hallucinations in the aged, cases that developed after ophthalmic surgery have been reported, but these patients were not included in the present study. Fifth, the presence of hallucinations may have been underestimated, as some patients experience little distress from hallucinations, and thus may not express them. In addition, due to the impairment in consciousness caused by delirium, the patients may not have been able to give a complete account of what they were experiencing or feeling.

In conclusion, regardless of these limitations, this is one of the largest studies to investigate factors affecting hallucinations in patients with delirium in a general hospital. The four factors found to affect hallucinations in the present study were not derived from physical or environmental factors, but rather, from the use of or withdrawal from exogenous substances. Although appropriate treatment differs depending on the etiological exogenous substance, hallucinatory symptoms that appear during delirium can be treated by modifying the medication.

## Data Availability

The data that support the findings of this study are available from the corresponding author upon reasonable request.

## References

[1] Inouye SK, Westendorp RG, Saczynski JS (2014). Delirium in elderly people. Lancet.

[2] Oh ES, Fong TG, Hshieh TT, Inouye SK (2017). Delirium in older persons: Advances in diagnosis and treatment. JAMA.

[3] American Psychiatric Association (2013). Diagnostic and Statistical Manual of Mental Disorder.

[4] Grover S, Shah R (2011). Distress due to delirium experience. Gen. Hosp. Psychiatry..

[5] Grover S, Ghosh A, Ghormode D (2015). Experience in delirium: is it distressing?. J. Neuropsychiatry. Clin. Neurosci..

[6] Schmitt EM (2019). Perspectives on the delirium experience and its burden: Common themes among older patients, their family caregivers, and nurses. Gerontologist..

[7] Trzepacz PT (2001). Validation of the Delirium Rating Scale-revised-98: Comparison with the delirium rating scale and the cognitive test for delirium. J. Neuropsychiatry. Clin. Neurosci..

[8] Meagher D (2007). Phenomenology of delirium: Assessment of 100 adult cases using standardised measures. Br. J. Psychiatry..

[9] Barodawala S, Mulley GP (1997). Visual hallucinations. J. R. Coll. Physicians. Lond..

[10] Ottens TH (2020). Hallucinations after cardiac surgery: A prospective observational study. Medicina.

[11] Ashton H (1997). Visual hallucinations. J. R. Coll. Physicians. Lond..

[12] Tondo L, Baldessarini RJ (2020). Discontinuing psychotropic drug treatment. BPsych. Open.

[13] Jobert A (2020). Benzodiazepine withdrawal in older people: What is the prevalence, what are the signs, and which patients?. Eur. J. Clin. Pharmacol..

[14] Uchida H (2009). Survey of benzodiazepine and antidepressant use in outpatients with mood disorders in Japan. Psychiatry. Clin. Neurosci..

[15] Uchida H (2009). Benzodiazepine and antidepressant use in elderly patients with anxiety disorders: A survey of 796 outpatients in Japan. J. Anxiety. Disord..

[16] Takeshima N, Ogawa Y, Hayasaka Y, Furukawa TA (2016). Continuation and discontinuation of benzodiazepine prescriptions: A cohort study based on a large claims database in Japan. Psychiatry. Res..

[17] Doane J, Stults B (2013). Visual hallucinations related to angiotensin-converting enzyme inhibitor use: Case reports and review. J. Clin. Hypertens..

[18] Aloisi G, Italian Study Group on Delirium (ISGoD) (2019). Drug prescription and delirium in older inpatients: Results from the Nationwide Multicenter Italian Delirium Day 2015–2016. J. Clin. Psychiatry..

[19] Farag E (2020). Association between use of angiotensin-converting enzyme inhibitors or angiotensin receptor blockers and postoperative delirium. Anesthesiology.

[20] Lizarraga KJ, Fox SH, Strafella AP, Lang AE (2020). Hallucinations, delusions and impulse control disorders in Parkinson disease. Clin. Geriatr. Med..

